# Fisetin Lowers *Streptococcus suis* serotype 2 Pathogenicity in Mice by Inhibiting the Hemolytic Activity of Suilysin

**DOI:** 10.3389/fmicb.2018.01723

**Published:** 2018-07-30

**Authors:** Yanyan Zhang, Bingbing Zong, Xiangru Wang, Yongwei Zhu, Linlin Hu, Pei Li, Anding Zhang, Huanchun Chen, Manli Liu, Chen Tan

**Affiliations:** ^1^Hubei Biopesticide Engineering Research Centre, Hubei Academy of Agricultural Sciences, Wuhan, China; ^2^State Key Laboratory of Agricultural Microbiology, College of Veterinary Medicine, Huazhong Agricultural University, Wuhan, China; ^3^Key Laboratory of Preventive Veterinary Medicine in Hubei Province, The Cooperative Innovation Center for Sustainable Pig Production, Wuhan, China; ^4^Key Laboratory of Development of Veterinary Diagnostic Products, Ministry of Agriculture of the People’s Republic of China, Wuhan, China; ^5^International Research Center for Animal Disease, Ministry of Science and Technology of the People’s Republic of China, Wuhan, China

**Keywords:** *Streptococcus suis* 2, pathogenicity, suilysin, hemolytic activity, anti-virulence compound, fisetin, infection

## Abstract

*Streptococcus suis* serotype 2 is a serious zoonotic pathogen and has attracted worldwide attention since the first human case was reported in Denmark in 1968. Some virulence factors have been reported to be involved in the pathogenesis of the infection caused by *Streptococcus suis* serotype 2, and then novel strategies to identify some anti-virulence compounds which can effectively inhibit the pathogenic bacterial infection have recently been reported. Suilysin is an essential virulence factor for *Streptococcus suis* serotype 2 since it creates pores in the target cells membranes, which aids bacterial colonization. The important role of suilysin in the virulence of *Streptococcus suis* serotype 2 renders it an ideal target for designing novel anti-virulence therapeutics. We find that fisetin, as a natural flavonoid, is a potent antagonist against suilysin-mediated hemolysis. The aim of this study is to evaluate the effect of fisetin on the hemolytic activity of suilysin from *Streptococcus suis* serotype 2. Fisetin is found to significantly inhibit the hemolytic activity of suilysin. Within the range of effective concentrations, fisetin does not influence the growth of *Streptococcus suis* serotype 2 and the expression of suilysin protein. *In vitro*, fisetin effectively inhibits the death of macrophages (J774A.1 and RAW264.7) infected with *Streptococcus suis* serotype 2 by weakening intracellular bacterial multiplication. Animal model experiment shows that fisetin effectively improves the survival rate of animals infected with *Streptococcus suis* serotype 2. Our findings suggest that fisetin could be used as an antitoxin against suilysin and be developed into a promising therapeutic candidate for treating *Streptococcus suis* serotype 2 infection.

## Introduction

*Streptococcus suis* (*S. suis*) is a common pathogen which can cause a variety of diseases, including meningitis, arthritis, septicemia, pneumonia, and endocarditis (Staats et al., 1997; Sriskandan and Slater, 2006; Kerdsin et al., 2016). These diseases cause a severe economic loss to the swine industry worldwide and pose a threat to human health. Among the 33 known serotypes of *S. suis*, serotype 2 (SS2) is the most prevalent serotype in pigs and humans and is frequently reported all over the world (Liu et al., 2013; Haas and Grenier, 2016). SS2 is an emerging zoonotic agent infecting humans and pigs and can cause meningitis, endocarditis, and streptococcal toxic shock-like syndrome (STSLS) in humans (Sriskandan and Slater, 2006). SS2 can infect people who are in contact with sick pigs, carrier pigs, or raw pork via wounds on the skin, or via oral or nasal mucosa (Francois et al., 1998). So far, more than 1642 cases of *S. suis* human infection have been reported worldwide (Guillaume et al., 2014).

To infect the host, *S. suis* must successfully pass through epithelial barriers, escape the host’s immune system, multiply in the bloodstream, and invade various organs, finally resulting in the necrosis of tissue cells or organs (Gottschalk et al., 2010; Fittipaldi et al., 2012; Li et al., 2013). A series of virulence factors, including capsular polysaccharide, muramidase-released proteins, suilysin, and fibronectin-binding proteins synthesized by *S. suis* play a key role in the infection process (Segura et al., 2004; Gottschalk et al., 2010; Fittipaldi et al., 2012). Therefore, these key virulence factors may be an ideal target for developing novel anti-virulence therapeutics to treat *S. suis* infection. Among these virulence factors, suilysin has been reported to exhibit hemolytic activity in different cell types (Du et al., 2013; Leung et al., 2014) and is considered a crucial factor for pathogenesis (Gottschalk and Segura, 2000).

Previous studies (Wu et al., 2011; Zhang et al., 2012) reported that SS2 strain SC19 can secrete suilysin encoded by the *sly* gene and is highly pathogenic to mice and pigs, causing STSLS. Suilysin is a well-known extracellular protein with a molecular weight of about 54 kDa (Fittipaldi et al., 2012). Suilysin belongs to the cholesterol-dependent cytolysin family, exhibiting cytotoxicity to epithelial cells, endothelial cells, neutrophils, macrophages (Charland et al., 2000; Lalonde et al., 2000; Segura and Gottschalk, 2002; Chabot-Roy et al., 2006), and antiphagocytic and antibactericidal properties in response to neutrophils and macrophages (Chabot-Roy et al., 2006; Benga et al., 2008; Fittipaldi et al., 2012). In addition, the mortality rate of mice infected with SS2 strain ST1 which produces high levels of suilysin can reach 90% within 10 days. SS2 Strain ST104 that produces only low levels of suilysin was found to have a lower pathogenicity in mice (Takeuchi et al., 2014). Other studies have also demonstrated that non-suilysin-synthesizing *S. suis* has lower virulence or is even avirulent in animal model (Staats et al., 1999; Lun et al., 2003; Takeuchi et al., 2014). Furthermore, studies show that SS2 virulence is largely dependent on suilysin expression (King et al., 2001; Takeuchi et al., 2014). Given all that, suilysin as a secreted protein is a critical virulence factor for SS2 to successfully colonize in host cells and escape the immune system of the host. Therefore, new anti-virulence compound which can effectively inhibit the hemolytic activity of suilysin could be novel therapeutic agent to treat SS2 infection.

Recently, several studies have reported that flavonoids including myricetin and morin hydrate can significantly weaken the virulence of SS2, and furthermore, the weakened pathogenicity of bacteria is due to the inhibition of hemolytic activity of suilysin (Li et al., 2017; Niu et al., 2017). Fisetin also belonging to flavonoids, as a potential antimicrobial compound, has been identified (Taechowisan et al., 2014). Moreover, Wang et al. reported that fisetin effectively inhibited *L. monocytogenes* virulence by inhibiting the hemolytic activity of Listeriolysin O (LLO) and exhibited little antimicrobial activity. Therefore, fisetin could be considered one of the promising and effective candidates to meet the challenge posed by widespread drug resistance to bacterial pathogens (Rasko and Sperandio, 2010).

In this study, the effect of fisetin on suilysin activity and the virulence of SS2 will be investigated. It is hypothesized that fisetin may effectively inhibit the hemolytic activity of suilysin, and that both *in vitro* and *in vivo*, fisetin will result in a significant decrease in the virulence of SS2. The findings may suggest that fisetin could be a potential therapeutic candidate for treating SS2 infection by inhibiting the hemolytic activity of suilysin.

## Materials and Methods

### Bacteria Strains, Growth Conditions, Fisetin Preparation

The SS2 strain SC19 used in this study is a virulent strain isolated from the brain of a dead pig during the epidemic outbreak in the Sichuan Province of China in 2005 (Li et al., 2013). The mutant Δ*sly* was constructed in SC19 using the thermosensitive suicide plasmid pSET4s (Takamatsu et al., 2001a), as described previously (Zhang et al., 2016). Its complementation strain SC19 Δ*sly::sly* was generated using the *Escherichia coli–Streptococcus suis* shuttle vector pSET2 (Takamatsu et al., 2001b), as described previously (Zheng et al., 2015). SC19 and Δ*sly* were grown in tryptic soy broth (TSB) or plated on tryptic soy agar (TSA) (Difco Laboratories, Detroit, MI, United States) with 10% newborn bovine serum added (Sijiqing Biological Engineering Materials Co., Ltd., Hangzhou, China) at 37°C. For western blot assay, the serum was heated to 56°C. After keeping temperature for 1 h, the serum was immediately cooled to 4°C and the denaturation BSA was removed by centrifugation for 15 min at 12,000 rpm at 4°C. After repeating seven times, BSA was detected in serum by enzyme-linked immunosorbent assay (CEA248Ge, Cloud-Clone Corp). Fisetin was purchased from Sigma-Aldrich and was dissolved in dimethyl sulfoxide (DMSO; Sigma-Aldrich, St Louis, MO, United States) to make a stock solution of various concentrations.

### Titration of Hemolytic Activity

The hemolytic activity of the SC19 supernatant was measured by the previously described methods with minor modifications (Jacobs et al., 1994). After SC19 was cultivated in TSB with additional 10% newborn bovine serum for 18 h at 37°C, the supernatant was harvested after centrifugation for 10 min at 10,000 rpm at 4°C. Two-fold serially diluted aliquots (150 μl) of test samples were added to 96-well cell culture clusters with phosphate-buffered saline (PBS) as the diluent. Then, 150 μl of PBS containing a 2% defibrinated sheep blood was added to each well. Subsequently, the plates were incubated on a Coulter mixer at 37°C. After incubation for 3 h, unlysed erythrocytes were removed through centrifugation for 10 min at 1,000 rpm at 4°C; then 150 μl aliquots of supernatant fluids were transferred to cuvettes and measured at the optical density of 543 nm (OD_543_) with BioSpectrometer (Eppendorf). The hemolytic activity of the supernatant with different concentrations to erythrocytes was measured. Samples treated with 1% Triton X-100 were set as 100% lysis control. Hemolysis was indicated by the ratio of OD_543_ of each sample to the complete lysis control.

### Effect of Fisetin on the Hemolytic Activity of Suilysin

To evaluate fisetin’s ability to inhibit hemolysis, hemolytic activity of the SC19 culture supernatant was measured as described previously with minor modifications (Smith-Palmer et al., 2002). Overnight cultures of SC19 were transferred into TSB (1:100) with additional 10% newborn bovine serum and different concentrations of fisetin (0, 2, 4, 8, 16, and 32 μg/ml). After incubation for 18 h at 37°C, the supernatant was collected after centrifugation for 10 min at 10,000 rpm at 4°C.

To evaluate the effect of fisetin to the hemolytic activity of suilysin in SC19 culture supernatant, SC19 without fisetin was cultured for 18 h at 37°C. Subsequently, SC19 supernatant was collected after centrifugation for 10 min at 10,000 rpm at 4°C. Different concentrations fisetin (0, 2, 4, 8, 16, and 32 μg/ml) were added to SC19 supernatant and incubated for 30 min at 37°C. Finally, the hemolytic activity of the collected supernatant which has been incubated with fisetin was measured according to the method described above.

### Anti-SC19 Activity of Fisetin Assay

The sensitivity of SC19 to fisetin was measured as previously described with minor modifications (Ding et al., 2015). The minimal inhibitory concentration (MIC) of fisetin against SC19 was determined by a serial dilution method, according to the procedures of the CLSI guideline M31-A2. Overnight cultures of SC19 in TSB with 10% newborn bovine serum were diluted into 10 ml aliquots at a density of 5 × 10^5^ CFU/ml, and a series of dilute concentration of fisetin (0, 2, 4, 8, 16, 32, 64, 128, 256 μg/ml) was added into cell culture plate. To measure the effect of fisetin (0, 2, 4, 8, 16, 32, 64, 128 μg/ml) on SC19, bacterial growth was monitored every 30 min at the optical density of 600 nm (OD_600_) by using an Automated Microbiology Growth Curve Analysis System (Bioscreen C), as previously described (Xu et al., 2014).

### Western Blot Assay

In order to investigate the changes of suilysin expression secreted into the SC19 supernatant during the course of the culture, the amount of suilysin in the culture supernatant was examined by western blot assay. SC19 in stationary phase were diluted (1:100) in 200 ml TSB with additional 10% newborn bovine serum at 37°C. Meanwhile, different concentrations of fisetin (0, 8, 16, 32 μg/ml) were added to the cultures. The mixture was then incubated at 37°C, and the culture supernatant was collected at 0, 4, 8, and 16 h. Before preparing the samples, the same amount of the purified BSA was added into different culture supernatants. It was followed by centrifugation for 10 min at 10,000 rpm at 4°C. The culture supernatants were mixed with SDS sample buffer, boiled for 10 min, and separated by 12% sodium dodecyl sulfate polyacrylamide gel electrophoresis. The protein was transferred to polyvinylidene difluoride membranes and suilysin was visualized by primary rabbit anti-*S. suis* suilysin antibody (bs-4537R) diluted at 1:1000 and secondary HRP-conjugated anti-rabbit IgG antibody (Danvers, MA, United States) diluted at 1:4000 in TBS with additional 5% skimmed milk powder, as described previously (Yi et al., 2017).

Exogenous addition of BSA as an internal control was visualized by primary mouse anti-BSA purified antibody (sc-57504) diluted at 1:800 and secondary HRP goat anti-mouse IgG antibody (AS003, abclonal) diluted at 1:5000 in TBS with additional 5% skimmed milk powder.

### Effect of Fisetin on SC19 Survival in Macrophage Cells

The intracellular survival assays in mouse J774A.1 macrophage-like cells and RAW264.7 macrophage cells were performed as described previously with minor modifications (Zhang et al., 2016). Mouse J774A.1 macrophage-like cells were grown in dulbecco modified eagle medium [DMEM, Invitrogen] with additional high glucose, minimum essential medium [MEM, Sigma], and 10% heat-inactivated fetal bovine serum [hiFBS] and RAW264.7 macrophage cells were cultured in DMEM with 10% hiFBS. Subsequently, both J774A.1 and RAW264.7 were maintained at 37°C in a humidified chamber with 5% CO_2_. Then 100 U/ml penicillin and 100 μg/ml streptomycin were added. The J774A.1 cells and RAW264.7 cells in antibiotic-free medium were seeded at 2 × 10^5^ cells per well in a 24-well tissue culture plate and incubated at 37°C with 5% CO_2_ until 80% confluency was achieved. Bacteria grown to exponential phase were suspended at 1 × 10^7^ CFU/ml in DMEM with or without fisetin (32 μg/ml). The 80% confluent monolayer cells were washed twice with PBS and the suspensions were distributed to 24-well plates (500 μl/well; MOI = 10). After coculture for 1 h, the suspensions were removed and the cells were washed three times with PBS and then exposed to DMEM containing 10% hiFBS containing gentamicin (100 μg/ml) and penicillin-G (5 μg/ml) with or without (32 μg/ml) for 1 h to kill extracellular bacteria. Afterward, the cells were washed again, and the fresh DMEM containing 10% of hiFBS with or without fisetin (32 μg/ml) was distributed to 24-well plates (1 ml/well). The number of viable bacteria associated with each sample was determined after 2, 4, and 6 h incubation at 37°C under 5% CO_2_.

### Lactate Dehydrogenase (LDH) Cytotoxicity Assay

The effect of fisetin on SC19 virulence was evaluated by LDH assay as previously described with minor modifications (Charland et al., 2000). J774A.1 cells were cultured in a medium at 37°C in a 5% CO_2_ atmosphere according to the method described above. J774A.1 cells were washed twice with PBS and resuspended to 10^5^ cells/ml in DMEM containing 10% of hiFBS in LDH release assay. Two hundred microliters of cell suspension was seeded per well in 96-well plates. After overnight culture, cells were washed twice with PBS and subsequently infected with bacteria which were grown to the mid-log phase and were resuspended in fresh DMEM containing various concentrations of fisetin (0, 2, 4, 8, 16, and 32 μg/ml). J774A.1 Cells treated with DMEM with or without 2.5% of Triton X-100 served as the positive and negative controls, respectively. After coculture for 5 h at 37°C, the supernatants were collected from 96-well plates by centrifugation (400 rpm, 5 min), and LDH released into supernatants was determined using the LDH Cytotoxicity Assay Kit (C0016, Beyotime, China).

### RNA Isolation, Quantitative PCR (qPCR), and Enzyme-Linked Immunosorbent Assays (ELISAs) for Cytokines

After J774A.1 cells infected SC19 were treated with fisetin, the expressions of TNF-α and IL-1β were measured by qPCR as reported previously (Liu et al., 2017). Briefly, the total RNA of cells was extracted using the TRIzol^®^ reagent (ambion). The RNA was used for cDNA synthesis and qPCR. The primers for the qPCR are listed in **Table 1**. β-actin was used as a reference gene. The protein expression of TNF-α and IL-1β in the cell culture supernatants were determined using commercially available ELISA kits (DAKEWE), following the manufacturer’s instructions.

**Table 1:** Primers used in this study.

Gene	Forward (5′–3′)	Reverse (5′–3′)	Species
IL-1β	CACTACAGGCTCCGAGATGA	CGTTGCTTGGTTCTCCTTGT	Murine
TNF-α	CCAGTCTGTATCCTTCTAA	TTGTGTTTCTGAGTAGTTG	Murine
β-actin	GGGAAATCGTGCGTGACAT	GCTCGTTGCCAATAGTGATGA	Murine


### Animal Experiments

To evaluate the effects of fisetin on SC19 virulence, 6-week-old female BALB/c mice were purchased from China Three Gorges University. Animal experimentation was approved by the local ethical committee, and all experiments were performed under institutional and national guidelines (HZAUMO-2017-019).

The animal experiments were performed as previously described with some modifications (Zong et al., 2016). SC19 in stationary phase was transferred to TSB (1:100) containing 10% of newborn bovine serum. After grown to mid-log phase (OD_600_ = 0.8) at 37°C, SC19 was harvested by centrifugation for 10 min at 6,000 rpm at 4°C, washed once with PBS and suspended in PBS.

Mice used for the survival rate study were injected via the tail vein with 100 μl bacterial suspension at a concentration of 2.5 × 10^9^ CFU ml. Mice injected with PBS containing no bacteria act a control. The group 2 h after infection with SC19 (12 per group) was first injected subcutaneously with fisetin at 100 mg/kg, and injected at 8 h intervals at the same dosage. The control group (12 per group) was injected with DMSO. The survival rate of mice was recorded every 12 h from 0 to 96 h after treatment. The body weight of mice was recorded every day from day 0 to day 14 after treatment.

Mice used for bacteria loads and pathological analysis were inoculated intravenously with a 100 μl bacterial suspension at a concentration of 2.5 × 10^8^ CFU ml. In the same method described above, fisetin was injected subcutaneously. At 36 h after infection, the number of bacteria in blood, brain, spleen, and liver was determined by grinding, diluting, and plating onto TSA containing 10% of newborn bovine serum. To examine pathological change caused by bacteria, the organs (the brain, spleen, and portions of liver) were fixed in 4% of paraformaldehyde for pathological examination. Mice in this study were euthanized with anesthesia followed by cervical dislocation.

## Results

### Fisetin Attenuates the Hemolytic Activity of Suilysin

**FIGURE 1:**
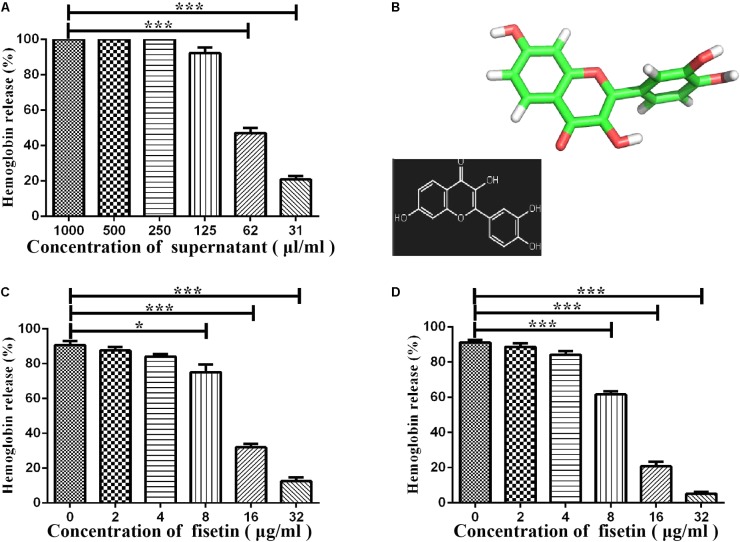
Inhibition of suilysin-induced hemolysis by fisetin: **(A)** The culture supernatant of SC19 cultures causes sheep erythrocyte hemolysis. Hemolysis was measured by hemoglobin release at OD_543_. **(B)** Chemical structure of fisetin. **(C)** After SC19 cultures were incubated with fisetin (0, 2, 4, 8, 16, and 32 μg/ml) at 37°C for 18 h, 125 μl culture supernatant was incubated with 875 μl of PBS containing a 2% defibrinated sheep blood at 37°C for 30 min. Fisetin significantly inhibited the hemolytic activity of the supernatant of SC19 cultured in TSB with 10% newborn bovine serum with 32 μg/ml fisetin. **(D)** After SC19 was cultured at 37°C for 18 h, 125 μl culture supernatant incubated with fisetin (0, 2, 4, 8, 16, and 32 μg/ml) at 37°C for 30 min was incubated with 875 μl of PBS containing a 2% defibrinated sheep blood at 37°C for 30 min. The inhibitor effect of fisetin on the suilysin in the supernatant of SC19 is dose dependent. Statistical analyses were performed using the two-tailed unpaired *t*-test. Statistically significant differences are indicated. ^∗^*P* < 0.05; ^∗∗^*P* < 0.01; ^∗∗∗^*P* < 0.001.

Some studies have reported that flavonoids can decrease the virulence of bacterial pathogens (Kang et al., 2006; Wang et al., 2015; Li et al., 2017; Niu et al., 2017) by inhibiting the hemolytic activities of hemolysin. It can be concluded that the suilysin in the SC19 culture supernatant could sufficiently induce hemolysis since approximately 95% of sheep erythrocytes were lysed at the concentration of supernatant (125 μl/ml) (**Figure 1A**). We found that fisetin (**Figure 1B**), a dietary flavonoid found in vegetables and fruits, significantly inhibited the hemolytic activity of a culture supernatant of SC19 in the stationary phase (**Figure 1C**). In addition, our further study found that the hemolytic activities of suilysin protein in the culture supernatant of SC19 were remarkably decreased in a dose-dependent manner after its co-culture with fisetin. Maximal inhibition was achieved at 32 μg/ml (**Figure 1D**). All these results point to the conclusion that fisetin decreases the hemolytic activity of suilysin.

### Fisetin Within the Effective Concentration Range Does Not Influence the Growth Characteristic of SC19

**FIGURE 2:**
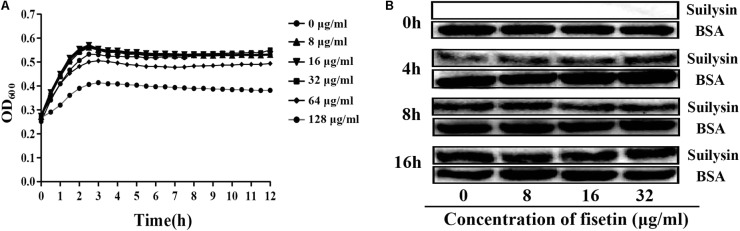
Fisetin at the concentrations of 32 μg/ml does not affect the growth characteristics of SC19. **(A)** SC19 was co-incubated with 0, 8, 16, 32, 64, 128 μg/ml fisetin in TSB containing 10% of newborn bovine serum, and the growth characteristic of SC19 was measured by OD_600_ every hour. **(B)** The effect of fisetin (0, 8, 16, 32 μg/ml) on the expression of suilysin in SC19 supernatant was investigated at 0, 4, 8, and 16 h by western blotting. Exogenous addition of BSA was detected as an internal control using an anti-BSA antibody. The medium supernatant without addition of SC19 was detected by western blot assay at 0 h.

To confirm that the decrease in hemolytic activity was not attributed to the change in normal growth of bacteria, we co-cultured SC19 with fisetin at the concentration of 0, 8, 16, 32, 64, and 128 μg/ml fisetin in TSB containing 10% of newborn bovine serum for 16 h. The results showed that fisetin (64 and 128 μg/ml) significantly inhibited the growth characteristics of SC19. However, fisetin (0, 8, 16, and 32 μg/ml) did not affect the growth characteristics of SC19 (**Figure 2A**).

To further confirm that the expression levels of suilysin is not altered by fisetin (0, 8, 16, and 32 μg/ml). Therefore, the amount of suilysin in the supernatant incubated with fisetin (0, 8, 16, and 32 μg/ml) at 0, 4, 8, and 16 h were measured by western blotting (**Figure 2B**). BSA was detected as an internal control using an anti-BSA antibody. Additionally, the MIC of fisetin against SC19 was greater than 128 μg/ml. These results indicated that fisetin at the concentration of 32 μg/ml did not affect SC19 growth or the normal expression of suilysin in the bacteriological medium. Taken together, our results suggest that fisetin inhibits the hemolytic activity of suilysin.

### Fisetin Attenuates Bacteria Proliferation in J774A.1 and RAW264.7 Macrophages

**FIGURE 3:**
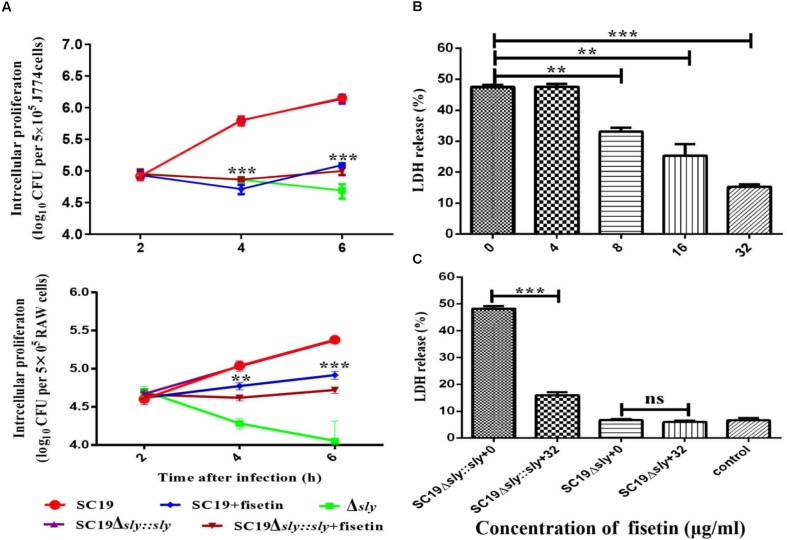
Fisetin inhibits intracellular colonization and cytotoxicity of SC19 in macrophages. **(A)** Fisetin inhibits intracellular bacterial growth in J774A.1 and RAW264.7 cells (MOI = 10). The results were expressed as means ± SD of recovered bacteria/ml. **(B)** The cytotoxicity of SC19 to J774A.1 cells is inhibited by fisetin in a dose-dependent manner. **(C)** J774A.1 cells infected with Δ*sly* and SC19Δ*sly::sly* were treated with 32 μg/ml fisetin. Statistical analyses were performed using the two-tailed unpaired *t*-test. Statistically significant differences are indicated. ^∗^*P* < 0.05; ^∗∗^*P* < 0.01; ^∗∗∗^*P* < 0.001.

The function of fisetin to effectively inhibit the hemolytic activity of suilysin without causing other growth changes has been confirmed above. Therefore, it can be speculated that fisetin could effectively inhibit intracellular bacterial growth in J774A.1 and RAW264.7 macrophages. As expected, SC19 level in infected J774A.1 cells treated with 32 μg/ml fisetin was decreased eight folds compared with that in infected cells without fisetin treatment. In addition, the deletion of *sly* gene caused bacterium number to significantly decrease, but the number bacteria that were complemented with *sly* gene was found not to decrease. The number of bacteria SC19Δ*sly::sly* in the cells treated with 32 μg/mL fisetin was also found to significantly decrease. These results suggest that fisetin can strikingly decrease bacterium number in macrophages (**Figure 3A**).

Some studies have reported (by LDH release) that SC19 is able to cause dramatic cytotoxicity (Charland et al., 2000). This study found that the cytotoxicity of SC19 to J774A.1 cells is inhibited by adding different concentrations of fisetin (**Figure 3B**). Furthermore, we found that 32 μg/ml fisetin can significantly decrease cytotoxicity and the function of fisetin is dose-dependent (**Figures 3B,C**).

### Inhibition of Intracellular SC19 Growth Is Attributed to Supplementation With Fisetin Rather Than the Increase of Inflammatory Cytokines

**FIGURE 4:**
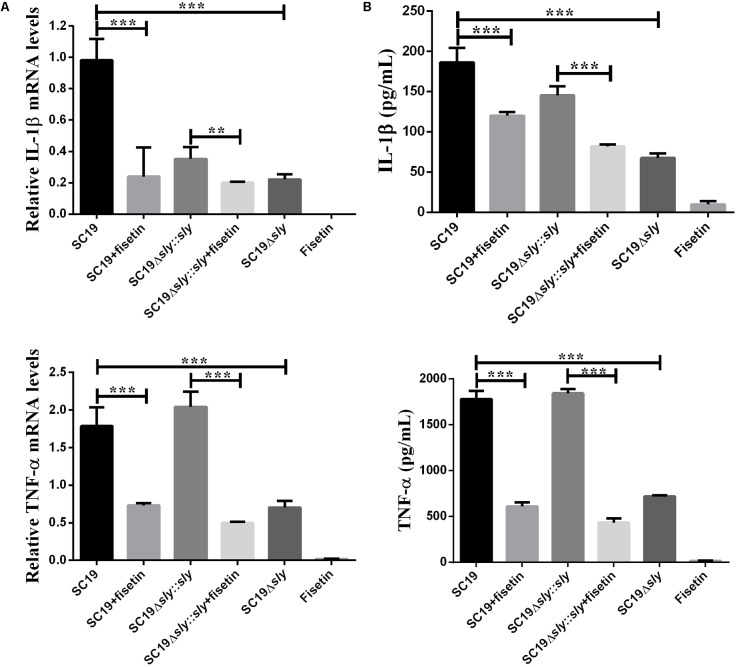
Induction of cytokine mRNA and protein expression in SC19-infected J774A.1 macrophages with or without fisetin treatment. J774A.1 macrophages were treated with 32 μg/ml fisetin in the absence (negative control) or presence of SC19 for 5 h. **(A)** The mRNA levels of IL-1β and TNF-α were determined by qRT-PCR. **(B)** The cytokine protein levels in the supernatants were determined by ELISAs. The bars represented the SEMs, on the basis of three independent experiments. Statistically significant differences are indicated. ^∗^*P* < 0.05; ^∗∗^*P* < 0.01; ^∗∗∗^*P* < 0.001.

After J774A.1 cells were incubated respectively with SC19, SC19+fisetin, SC19Δ*sly*::*sly*, Δ*sly*, or only fisetin, the expression levels of TNF-α and IL-1β were measured by qPCR. The SC19 and complementary SC19Δ*sly*::*sly* without additional fisetin led to the significant increase in the expression levels of TNF-α (**Figure 4A**) and IL-1β (**Figure 4B**) in J774A.1 cells, whereas SC19+fisetin or the deletion of *sly* gene significantly led to the decrease in the expression levels of TNF-α and IL-1β in J774A.1 cells. These results revealed that fisetin did not stimulate J774A.1 cells to release TNF-α and IL-1β, and that it remarkably inhibited the release of inflammatory factor in J774A.1 cells infected with SC19.

### Fisetin Lowers the Virulence of SC19 in Mouse Model

To determine the contribution of fisetin to the infected mice, SC19, Δ*sly* and SC19Δ*sly*::*sly* were used to infect mice. Twelve BALB/c mice in each group were infected via intravenous injection with 2.5 × 10^8^CFU bacteria or PBS (control) and monitored for examining their survival rate over a 4-day period. Intravenous injection of 2.5 × 10^8^CFU of SC19 made the fur of mice ruffle immediately. After injection, approximately 60% of infected mice were found to be killed by SC19 within 36 h. All infected animals died within 96 h. Conversely, 100% of Δ*sly*-infected mice were still alive and showed no infection-associated morbidity such as wasting or ruffling of fur within 96 h. However, SC19Δ*sly*::*sly* complemented with *sly* gene recovers its virulence. The infected mice exhibited ruffled fur immediately after infection and began dying at 12 h after infection. Only 8% survived within 96 h.

**FIGURE 5:**
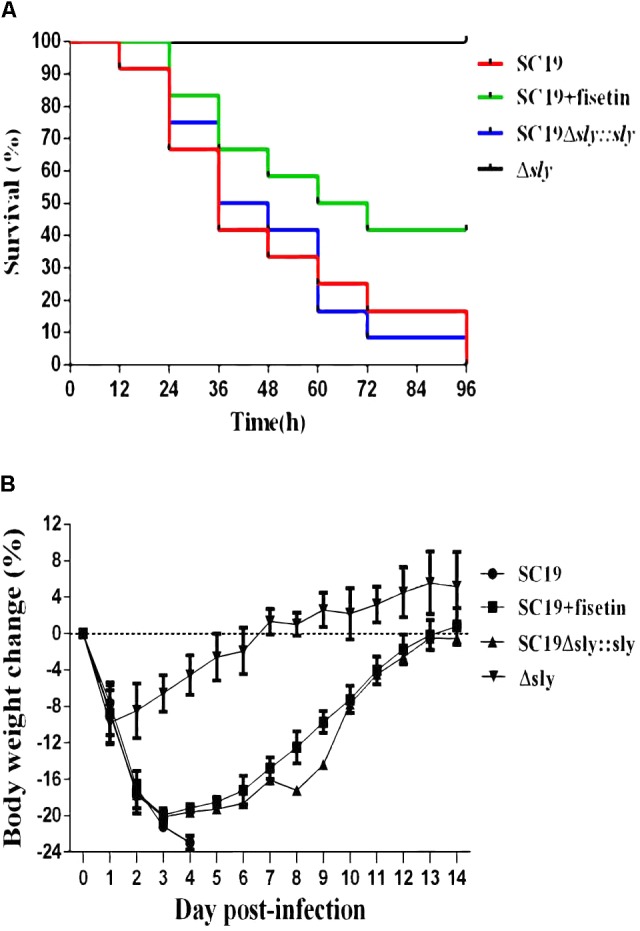
Fisetin treatment contributes to the survival of infected mice. **(A)** The effects of fisetin on the survival of mice infected with SC19, and the percentage of mice survival each day. *P* < 0.001 for comparison of the Δ*sly* group with the SC19Δ*sly*::*sly* group, and P < 0.001 for comparison of the SC19 group with SC19 + fisetin group. Mice injected with PBS as a control are not shown (the log-rank test). **(B)** The effect of fisetin on body weight changes of BALB/c mice infected with SC19. The average body weight change of surviving mice was calculated each day. Error bars represented SD from at least three independent experiments.

As expected, when fisetin at the concentration of 100 mg/kg was used to treat mice infected with SC19, 66.66 and 41.66% were alive within 36 and 96 h, respectively (**Figure 5A**). Mouse body weight was also monitored during the entire experiment period, and the results exhibited that SC19-infected and SC19Δ*sly::sly*-infected mice suffered from a severe weight loss after infection. However, Δ*sly*-infected mice experienced first a slight weight loss, and then started a weight gain 1 day after infection. The body weight of SC19-infected and fisetin-treated mice was observed to decrease by 20% within 3 days after infection and to increase from day 4 (**Figure 5B**).

### Fisetin-Treated Mice Exhibit the Resistance to SC19 Infection

**FIGURE 6:**
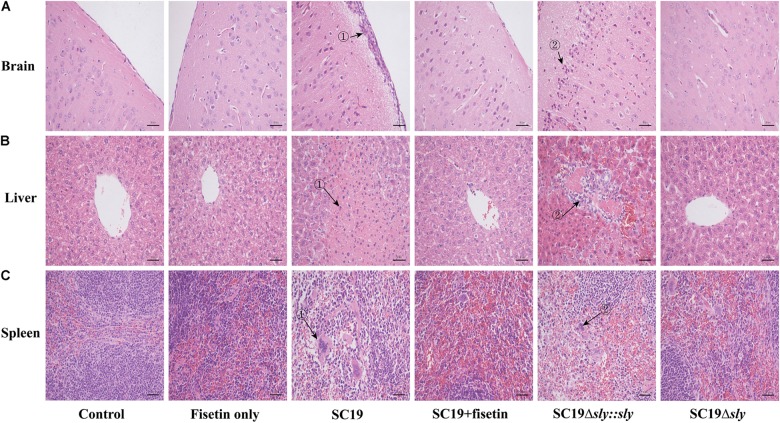
The effect of fisetin on the pathogenicity of SC19. Mice were injected intravenously with 2.5 × 10^7^ CFU of SC19, Δsly, and SC19Δ*sly::sly*, respectively. At 2 h after infection, the group infected with SC19 (12 per group) were injected subcutaneously with 100 mg/kg fisetin at 8 h intervals at the same dosage. The control group (12 per group) was injected with DMSO. At 36 h after infection, the organs (the brain, spleen, and portions of liver) were fixed in 4% of paraformaldehyde for pathological examination. Pathological examination of brain **(A)**, liver **(B)**, and spleen **(C)** tissues of the infected mice.

To evaluate the effect of fisetin on the pathogenicity of SC19 to the host, mice injected with a sublethal dose of bacteria (2.5 × 10^7^ CFU) were treated with or without fisetin. At 36 h after infection, the analyses of brains from mice untreated with fisetin exhibited severe congestion. In addition, livers and spleens showed organomegaly, severe congestion, and numerous small white necrotic foci. In fisetin-treated mice, visible lesions were hardly found with only mild congestion observed. Consistent with these findings, the brain tissues of the infected mice were severely thickened as the major histopathological characteristics (**Figure 6A**-➀) and a large number of inflammatory cells (**Figure 6A**-➁) were observed, whereas tissues from fisetin-treated mice bore few inflammatory cells.

Significant fatty infiltration (**Figure 6B**-➁) and numerous spotty necroses (**Figure 6B**-➁) were observed in the livers from the infected mice, whereas livers from fisetin-treated mice displayed only a few inflammatory lesions. Lymphocyte depletion and necrosis with congestion (**Figure 6C**-➀➁) were observed in germinal centers in the spleens from the infected mice, whereas only the mild inflammation was observed in the spleens from fisetin-treated mice.

Mice infected with Δ*sly* showed few pathological changes, which was consistent with these observations in SC19-infected and fisetin-treated mice. When gene *sly* was added into Δ*sly*, the pathogenicity of SC19Δ*sly::sly* was comparable to that of SC19 (**Figure 6**). Taken together, these results indicate that fisetin decreases the pathogenicity of SC19.

### Fisetin Decreased the Ability of SC19 to Colonize in Various Tissues of Mice

**FIGURE 7:**
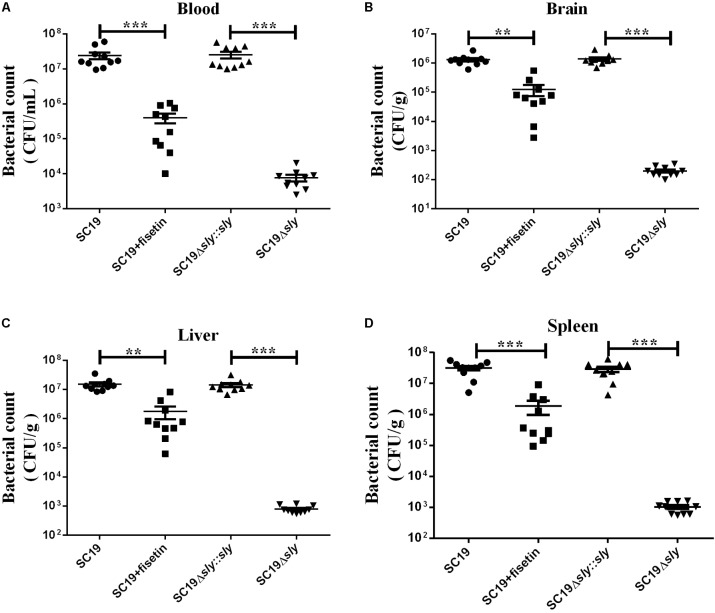
Bacterium number of SC19, Δ*sly*, and SC19Δ*sly::sly* in mice treated with or without fisetin. Mice were injected intravenously with 2.5 × 10^7^ CFU of SC19, Δsly, and SC19Δ*sly::sly*, respectively. Bacterium number in the blood **(A)**, brain **(B)**, liver **(C)**, and spleen **(D)** was counted at 36 h after infection. Statistical analyses were performed using the two-tailed unpaired *t*-test. Statistically significant differences are indicated. ^∗^*P* < 0.05; ^∗∗^*P* < 0.01; ^∗∗∗^*P* < 0.001.

Next, we investigated the effect of fisetin on bacterium number of SC19 in various tissues of mice injected with a sublethal dose of bacteria. Consistent with the multiplication of bacteria in the macrophages treated with or without fisetin (**Figure 3A**), dramatic differences in the multiplication of SC19 between fisetin-treated mice and untreated mice were observed at 36 h after infection. The bacterium number of SC19 in the blood (**Figure 7A**), brain (**Figure 7B**), liver (**Figure 7C**), and spleen (**Figure 7D**) of fisetin-treated mice was dramatically lower than that of mice untreated with fisetin. These results suggested that SC19 is more effectively eliminated in mice treated with fisetin than in untreated mice. In addition, the bacterium number of Δ*sly* was significantly smaller than that of SC19Δ*sly::sly*. The results above suggests that fisetin decreases the bacterium number of SC19 in mice by inhibiting the hemolytic activity of suilysin.

## Discussion

The mechanism of conventional antibiotics is to disrupt the essential functions of bacteria, such as cell-wall synthesis, DNA replication, or protein synthesis (Rasko and Sperandio, 2010). However, with the increase in antibiotic resistance of many clinically relevant bacteria, new antibacterial classes unaffected by resistance mechanisms must be urgently developed (Alanis, 2005; Terzulli et al., 2007). Drugs targeting virulence factors is an alternative approach to treat infections caused by resistant bacteria (Escaich, 2008). Moreover, anti-virulence factors have advantages over traditional antibiotics in two key ways. First, target genes that are essential for basic metabolism can be inhibited. These genes often exert essential functions in host/pathogen interactions and allow bacterial multiplication in the host. Second, the specificity of drugs targeting virulence factors could preserve the bacteria of the normal flora (Escaich, 2008). With the development of genomics and the availability of diverse *in vivo* gene expression technologies (Benton et al., 2004; Begun et al., 2005; Kurz and Ewbank, 2007), more mechanisms underlying bacterial infection are expected to be understood (Freiberg and Brötz-Oesterhelt, 2005; Burrack and Higgins, 2007).

Cytotoxins have been reported to be present in the diverse species of gram-positive bacteria. Cytotoxins such as intermedilysin expressed by *Streptococcus intermedius* (Nagamune et al., 1996), perfringolysin O expressed by *Clostridium perfringens* (Shepard et al., 1998), and listeriolysin O expressed by *Listeria monocytogenes* (Wang et al., 2015) are essential for the bacteria to successfully infect the host (Xu et al., 2010). Moreover, listeriolysin O has been adequately studied in mice models as a therapeutic target (Wang et al., 2015).

Consistent with previous reports, this study reveals that suilysin is an essential virulence factor for SC19 (**Figure 5A**). Suilysin is secreted into the extracellular space, leading to the lysis of host cells (Xu et al., 2010). Therefore, suilysin is a potential target to develop a new anti-suilysin compound which may attenuate SC19 pathogenicity without changing bacterial growth characteristics.

Recently, several studies have reported that flavonoids significantly weakened the virulence of pathogenic bacteria by inhibiting the activities of some important virulence factors (Kang et al., 2006; Wang et al., 2015). This study discovered that as a bioactive flavonoid molecule, fisetin (3, 3′, 4′, 7-tetrahydroxyflavone, **Figure 1A**) from fruits and vegetables (Ye et al., 2009), is found to effectively inhibit SC19 virulence in both tissue culture and animal model infection by inhibiting the hemolytic activity of suilysin.

*In vitro*, this study finds that fisetin efficiently inhibits the hemolytic activity of suilysin (**Figure 1C**), and that it has little antibacterial activity against SC19 at the concentrations of 32 μg/ml (**Figure 2A**). The result of our study is consistent with that of the experiment by Niu et al. (2017) who reported that myricetin inhibited suilysin cytotoxicity without exhibiting antimicrobial activity. Meanwhile, Li et al. (2017) reported that another flavonoid morin hydrate protects mice from SS2 infection by inhibiting the hemolytic activity of suilysin. Although both Wang et al. and Loose et al. reported that fisetin protected against *Listeria monocytogenes*, they showed contradictory results on the effect of fisetin on LLO activity. Wang et al. (2015) showed that fisetin inhibited the hemolytic activity of LLO, whereas Loose et al. (2016) demonstrated that fisetin inhibited LLO’s expression through PrfA rather than its hemolytic activity. In order to further investigate the effect of fisetin on hemolytic activity of SC19, the sequence of gene *prfA* from *Listeria monocytogenes* were compared with complete genome sequences of SC19 by NCBI BLAST. However, similar gene is not found in SC19. In addition, we found that the hemolytic activity of supernatant incubated with fisetin was inhibited (**Figure 1D**), and that there was no significant difference in suilysin expression level of SC19 with 32 μg/ml of fisetin (**Figure 2B**). Taken together, fisetin inhibits the hemolytic activity of SC19 as described by Li et al. (2017) and Niu et al. (2017).

Some recent reports show that serious bacteremia, high serum proinflammatory cytokine levels, and STSLS can be caused by SC19 (Ye et al., 2009; Bi et al., 2014). Suilysin is an important factor in STSLS (Wu et al., 2011; Zhang et al., 2012). We found that fisetin decreased the proinflammatory ability, bacterial loads, and lethality of SC19 in mice. These results strongly suggest that fisetin could be an effective therapeutic compound.

Previous studies confirms that inflammatory responses are usually beneficial to the host (Hersh et al., 1998). However, excessive inflammation is harmful and it can lead to shock and organ failure (Kahn et al., 2008). Some findings reveals that SS2 has evolved to acquire the ability to stimulate the host immune system to produce a large number of pro-inflammatory cytokines, such as TNF-α, IFN-γ, IL-1β, IL-6, IL-12, and MCP-1 (Dominguez-Punaro et al., 2008). Therefore, seeking and developing anti-inflammatory compounds will be essential for treating bacterial infections. As predicted, fisetin treatment significantly decreased the multiplication capacity of SC19 in macrophages (**Figure 3A**) and decreased SC19-induced macrophage injury according to a cytotoxicity assay (**Figure 3B**). This study further revealed that fisetin strikingly decreased the number of inflammatory cytokines such as IL-1β and TNF-α in cell models (**Figure 4**).

*In vivo*, the therapeutic effect of fisetin on mice infected with SC19 was evaluated. The survival rate of mice treated with 100 mg/kg fisetin was significantly higher than that of mice untreated with fisetin (**Figure 5A**). Some previous studies indicated that cytokine overexpression can break this balance, resulting in organ injury and speeding up disease progression (Tisoncik et al., 2012). Therefore, decreasing bacterial loads and the release of inflammatory factors can be an effective treatment. As expected, the multiplication capacity of SC19 was significantly decreased in the blood, brain, spleens, and livers of the infected mice treated with 100 mg/kg fisetin compared to that of the infected mice untreated with fisetin (**Figure 7**). In addition, the brain, spleen, and liver of the infected mice treated with 100 mg/kg fisetin showed few histopathological lesions than those of infected mice untreated with fisetin (**Figure 6**).

## Conclusion

Our results have demonstrated that fisetin can be a novel and effective compound to prevent and treat SC19 infection. However, there is much room for further study. *In vitro*, the inhibitory rate of fisetin on the hemolytic activity of suilysin can reach >85%. However, *in vivo*, the survival rate of infected mice treated with fisetin approximates 40%. Therefore, further research could improve the therapeutic efficacy of fisetin in clinical application by optimizing the structure and dosage of fisetin and changing the treatment route or using fisetin in combination with other antibiotics.

## Ethics Statement

This study was carried out in strict accordance with the Guide for the Care and Use of Laboratory Animals Monitoring Committee of Hubei Province, China, and the protocols and procedures were approved by the Committee on the Ethics of Animal Experiments at the College of Huazhong Agricultural University (Permit No. HZAUMO-2017-019).

## Author Contributions

YYZ and ML: performed the experiments mainly and some experiments were performed with the assistance of BZ, YWZ, LH, PL. YYZ and ML: analyzed the data. CT, HC, AZ, and XW: conceived and designed the study. YYZ and CT: wrote the manuscript.

## Conflict of Interest Statement

The authors declare that the research was conducted in the absence of any commercial or financial relationships that could be construed as a potential conflict of interest.
